# Drought effects on the tissue- and cell-specific cytokinin activity in poplar

**DOI:** 10.1093/aobpla/plx067

**Published:** 2017-12-01

**Authors:** Shanty Paul, Henning Wildhagen, Dennis Janz, Andrea Polle

**Affiliations:** Forest Botany and Tree Physiology, University of Goettingen, Büsgenweg, Göttingen, Germany

**Keywords:** Cambium, cytokinin, soil water deficit, type-A response regulator, wood

## Abstract

Climate change with increasing periods of drought is expected to reduce the yield of biomass crops such as poplars. To combat yield loss, it is important to better understand the molecular mechanisms that control growth under drought. Here, the goal was to resolve the drought-induced changes of active cytokinins, a main growth hormone in plants, at the tissue level in different cell types and organs of poplars (*Populus* × *canescens*) in comparison with growth, biomass, leaf shedding, photosynthesis and water potential. Since cytokinin response is mediated by type-A response regulators, *ARR5::GUS* reporter lines were used to map cytokinin activity histochemically. The expression of *PtaRR3* and *PtaRR10* was examined in different stem sections. Young leaves showed strong cytokinin activity in the veins and low staining under drought stress, accompanied by diminished leaf expansion. Leaf scars, at positions where drought-shedding occurred, showed strong reduction of cytokinin activity. The pith in the differentiation zone of stem showed high cytokinin activity with distinct, very active parenchymatic cells and enhanced activity close to primary xylem. This pattern was maintained under drought but the cytokinin activity was reduced. Mature phloem parenchymatic cells showed high cytokinin activity and mature wood showed no detectable cytokinin activity. Cytokinin activity in the cambium was apparent as a clear ring, which faded under drought. Xylem-localized cytokinin activities were also mirrored by the relative expression of *PtaRR3*, whereas *PtaRR10* showed developmental but no drought-induced changes. Primary meristems exhibited high cytokinin activity regardless of drought stress, supporting a function of this phytohormone in meristem maintenance, whereas declining cytokinin activities in apical pith tissues and cambium of drought-stressed poplars linked cytokinin in these cell types with the control of primary and secondary growth processes. Changes in cytokinin activity further imply a role in drought avoidance mechanisms of poplars, especially in the reduction of leaf area.

## Teaser

Cytokinins are important phytohormones that regulate plant growth. Here we localized cytokinin activities in poplar trees, which are important biomass crops. We demonstrated that even mild drought stress strongly suppresses cytokinin activities in the cambium, the "stem cells" of trees, resulting in loss in productivity and reduced wood formation. Cytokinin activities also decline in the leaf stalks before shedding. Our results identified critical tissues for drought acclimation in the woody biomass crop poplar and provide a basis for selection of climate-smart trees.

## Introduction

Cytokinins form a group of phytohormones that have roles in promoting cell division, shoot initiation, growth and the regulation of vascular development (Hwang *et al.* 2012; Kieber and Schaller 2014). Root tips are the major synthesis sites of cytokinins (Dieleman *et al.* 1997; Miyawaki *et al.* 2004; Aloni *et al.* 2005), which are then acropetally transported in the xylem sap by the transpirational pull to the above-ground tissues (Aloni *et al.* 2005). Active forms of cytokinins in plants are present in the form of free bases, whereas inactive forms are present as ribotides, ribosides or glucose conjugates (Mok and Mok 2001; Romanov *et al.* 2006).

Cytokinin perception and signalling in plants involves a His-Asp phosphorelay mediating the transmission of the signal (Mizuno 2005; Schaller *et al.* 2008). In this pathway, type-A *ARRs* (*Arabidopsis* response regulators) are the primary response genes for cytokinins in *Arabidopsis* (D’Agostino *et al.* 2000). Expression of an *Arabidopsis ARR5::GUS* reporter construct in poplar indicated conserved functions of the response regulator in both species (Paul *et al.* 2016). A total of 10 type-A *RR* genes are present in *Arabidopsis* (D’Agostino *et al.* 2000; Schaller *et al.* 2008; Pils and Heyl 2009) and 11 were reported in *Populus trichocarpa* (Ramírez-Carvajal *et al.* 2008; Immanen *et al.* 2013). The expression of these genes is transcriptionally regulated and induced by exogenous cytokinin (Taniguchi *et al.* 1998; D’Agostino *et al.* 2000; Paul *et al.* 2016).

Cytokinins play important roles in response to various abiotic stresses. Among the abiotic stresses faced by the plants, drought stress is of serious concern because periods of drought stress are likely to increase along with the rising global temperature in many areas of the world (IPCC 2007). Plants respond to environmental constraints by physiological and morphological adjustments such as decreases in stomatal conductance and reduced plant growth. Since cytokinins are negative regulators of root meristem activity and positive regulators of shoot meristem activity (Werner *et al.* 2003), it is conceivable that growth reduction under drought involves altered phytohormone levels. For example, a decreased content of cytokinins was found in alfalfa under drought (Goicoechea *et al.* 1996) which was accompanied by accelerated leaf senescence. The xylem sap of sunflower contained decreased cytokinin concentrations under drought (Bano *et al.* 1994; Shashidhar *et al.* 1996; Hansen and Dörffling 2003). Because drought-stressed plants often show increased root production and decreased shoot growth, it has been suggested that enhanced drought tolerance can be achieved by decreasing cytokinin levels through overexpression of systemic or root-specific cytokinin-degrading enzymes (Werner *et al.* 2010; Nishiyama *et al.* 2011; Mackova *et al.* 2013). In contrast to this proposal, rice (Peleg *et al.* 2011), tobacco (Rivero *et al.* 2007), peanut (Qin *et al.* 2011) and cotton plants (Kuppu *et al.* 2013) transformed with the *Agrobacterium IPT* gene, i.e. a cytokinin biosynthetic gene, under a stress inducible (SARK) promoter resulted in enhanced drought tolerance. These findings show that cytokinins play an important role in drought susceptibility although the underlying mechanisms are not yet fully understood (Peleg and Blumwald 2011; Zwack and Rashotte 2015).

Poplars are water-spending species that show strong changes in wood anatomy in response to drought and usually shed their leaves under dry conditions (Bogeat-Triboulot *et al.* 2007; Beniwal *et al.* 2010; Hennig *et al.* 2015). Whether these morphological alterations are accompanied by changes in cytokinin activity is not yet known. The aim of this study was to examine the occurrence and localization of active cytokinins in response to drought in different tissues of poplar (*Populus* × *canescens*) including shoot apices, leaves, leaf scars, stem and roots. Localization of active cytokinins in different cell types at different stem positions was also examined. We hypothesized that cytokinin activity shows a decrease in all plant parts, except in the meristems, when poplars suffer drought stress. Wild-type (WT) and transgenic *Populus × canescens* transformed with *ARR5::GUS* (β-glucuronidase) reporter gene construct (Paul *et al.* 2016) were used here to examine the localization pattern of active cytokinins. Cytokinin activity traced by the *ARR5::GUS* gene construct reflects cytokinin responses. In addition, the expression levels of two poplar response regulators, *PtaRR3* and *PtaRR10* (Pta stands for the hybrid *Populus tremula* × *Populus alba*, syn. *P.* × *canescens*), were also analysed at different stem positions. Drought stress was characterized by soil moisture content, photosynthesis and predawn leaf water potential of the plants.

## Methods

### Plant cultivation

Transgenic *Populus* × *canescens* transformed with *ARR5::GUS* reporter gene construct that were described by Paul *et al.* (2016) were used in this study. The *ARR5::GUS* poplar reporter lines 64 and 80 were *in vitro* micropropagated according to Leple *et al.* (1992). The transgenic plantlets along with the WT were grown for 3 weeks in hydroponic systems and afterwards transferred to pots (3 L) as described in Müller *et al.* (2013) with the difference that a 1:1 mixture of soil (Fruhstorfer Erde Type N, Hawite Gruppe GmbH, Vechta, Germany) and sand (one part coarse sand Ø 0.71–1.25 mm and one part fine sand Ø 0.4–0.8 mm; Melo, Göttingen, Germany) was used. Slow-release fertilizer pellets (2 g L^−1^ of soil volume; Nutricote T100, 13:13:13 NPK and micronutrients; FERTIL S.A.S., Boulogne Billancourt, France) were added to the soil-sand mixture at the time of planting. An amount of 2 g L^−1^ of soil volume of these pellets was added again to each pot, after one and a half months of plant growth. The plants were grown in a climate chamber under controlled environmental conditions: day/night length of 16/8 h, photosynthetically active radiation (PAR) of 150 µmol quanta m^−2^ s^−1^ (EYE Clean-Ace metal-halide lamps: 400 W, MT400DL/BH, EYE Iwasaki Electric Co. Ltd, Tokyo, Japan), 23 °C air temperature and 60 % relative air humidity. There were 14 biological replicates for each line.

### Drought treatment and measurement of physiological parameters

The plants were grown under well-watered conditions for 45 days. Then (Day 0), plants were divided into two groups (seven biological replicates per line and treatment) for application of two water levels: well-watered (control) plants, which received 250 mL tap water day^−1^ (125 mL each in the morning and evening) and drought-treated plants, for which water supply was gradually decreased during 2 weeks to a minimum level of 70 mL day^−1^ (35 mL each in the morning and evening). The reduced water supply was maintained for another 3 weeks. Soil moisture was measured after 10 min of watering using a ThetaProbe ML2X soil moisture sensor equipped with a HH2 readout unit (Delta-T Devices Ltd, Cambridge, UK). The well-watered and drought-treated plants of WT and transgenic lines were placed in a mixed design inside the climate chamber and randomized regularly.

During the treatment period, plant height was recorded every third day. The stem diameter (2 cm above the root collar) was recorded once a week. These measurements were conducted on all experimental plants. Predawn leaf water potential, net photosynthetic CO_2_ assimilation and transpiration were measured once a week. For the determination of the predawn leaf water potential, one leaf was removed from the middle of the plant stem, immediately installed in a Scholander pressure chamber (Soil-moisture Equipment Corp., Santa Barbara, CA, USA) and exposed to increasing pressure until the xylem sap appeared at the cut surface of the petiole. The measurements were performed during 1 h before the lights in the climate chamber were switched on. Three biological replicates per line and treatment were used to avoid overly damage to the plants.

For the gas exchange measurements, the first fully expanded leaf from the stem apex was selected. The measurements were performed with a portable photosynthesis system (LCPro+, ADC BioScientific Ltd, Hoddesdon, UK) with an additional light of 800 µmol quanta m^−2^ s^−1^ PAR, at a temperature of 23–25 °C (*T*_leaf_) and a relative air humidity of 60 %. Each plant was allowed to acclimatize to the additional light for 5 min. Then five measurements were recorded per leaf. Gas exchange was measured on five biological replicates per line and treatment because these samples could be measured within 3 h to avoid confounding effects of diurnal fluctuations.

### Harvest

All plants were harvested when the drought-treated plants showed the mean predawn leaf water potential of −0.85 ± 0.003 MPa. Leaves, stem, coarse roots and fine roots were separated during the harvest. In preliminary tests, root diameters were measured and for the experiments first- and second-order root branches whose diameters were less than 2 mm were cut and defined as fine roots. Fresh mass of each fraction was determined. In order to measure the dry mass, subsamples of each plant tissue were dried in an oven at 60 °C for 7 days. Dry mass of the leaves shed by the plants during the treatment period was recorded separately. Tissue dry mass (*M*_dt_) in grams was calculated as:

$$M_{\text{dt}}=(M_{\text{da}}\times M_{\text{ft}})/M_{\text{fa}}$$

where, *M*_da_ is the dry mass of the aliquot (g), *M*_ft_ is the total tissue fresh mass (g) and *M*_fa_ is the fresh mass of the aliquot (g).

During the harvest, the following fresh tissues were used for GUS staining: one half of the shoot apex, leaf disks (diameter 5 mm) from young leaves (not fully developed leaves which were seen just below the stem apex), stem cross-sections (2 mm thickness) from three positions: top (50 mm beneath the stem apex), middle (the position in the stem exactly in the centre between the apex and the shoot–root junction) and bottom (50 mm above the root–shoot junction), a leaf scar (by removing the lowest fresh leaf) and fine root tips. The plant materials were directly transferred into the staining solution for the GUS activity assay as described by Paul *et al.* (2016). Briefly, the tissues were vacuum-infiltrated with GUS buffer (100 mM sodium phosphate buffer, pH 7.0, 10 mM Na_4_EDTA, 0.05 % Triton X-100) containing 1 mg mL^−1^ 5-bromo-4-chloro-3-indolyl-β-D-glucuronic acid (Duchefa, Haarlem, The Netherlands). Afterwards, the tissues were incubated in darkness at 37 °C overnight, chlorophyll was removed by ethanol and the tissues were stored for further analyses.

To measure the leaf area, three leaves (from top, middle and bottom positions of stem) were collected from each plant and weighed. Leaves were scanned (Canoscan 4400F, Canon Inc., China) and leaf area was measured using Image J (Abramoff *et al.* 2004). Area per leaf of each plant was expressed as an average of the leaf area of leaves collected from three different positions. The whole-plant leaf area (*A*_t_) in cm^2^ was calculated as:

$$A_{\text{t}}=\;(M_{\text{ft}}\times A_{\text{a}})/M_{\text{fa}}$$

where, *M*_ft_ is the total leaves fresh mass (g), *A*_a_ is the sum of area of three leaves (cm^2^) and *M*_fa_ is the fresh mass of three leaves selected for leaf area (g).

During the harvest, stem pieces (without bark) from two different positions (top and bottom) were shock-frozen in liquid nitrogen and stored at −80 °C for molecular analyses.

### GUS activity analyses at tissue level and in different cell types in stem tissues

Documentation of the tissue-level staining patterns, the subsequent embedding of plant tissues in Technovit 7100 resin (Heraeus Kulzer GmbH & Co. KG, Germany) and sectioning of the embedded tissues were carried out as described in Paul *et al.* (2016). Sections of 20 μm thickness were used for analysis of active cytokinin localization in different cell types as reported by Paul *et al.* (2016).

### RNA extraction and cDNA synthesis

Frozen stem samples (without bark) from top and bottom positions were used to extract RNA. Four biological replicates per line, stem position and treatment were used. Each sample was ground in a ball mill (Retsch MM 400, Haan, Germany) in liquid nitrogen. An amount of ~150 mg of the tissue powder was used for RNA extraction after Chang *et al.* (1993) with modifications as described in Janz *et al.* (2012). Total RNA concentration was measured at a wavelength of 260 nm and quality was analysed based on the 260/280 and 260/230 ratios using a NanoDrop™2000 spectrophotometer (Thermo Scientific, Waltham, MA, USA). RNA integrity was confirmed by an agarose gel electrophoresis. DNA contamination was removed by treating the samples with Ambion® Turbo DNA-free™ kit (Life Technologies, Carlsbad, CA, USA) according to the instructions in the manual. RNA (1 µg) was transcribed to cDNA with First Strand cDNA Synthesis Kit (Thermo Fisher Scientific, Braunschweig, Germany) using random primers provided in the kit.

### Quantitative real-time PCR

Gene-specific primers were designed for the *P. × canescens* orthologs of Potri.002G082200 (*PtaRR3*), Potri.015G070000 (*PtaRR10*) with the Oligo Explorer (Gene Link, Hawthorne, NY, USA; http://www.genelink.com/). For this purpose, *P.* × *canescens* transcript sequences (coding sequences and untranslated regions) of all type-A RR genes (as listed in Paul *et al.* 2016) were obtained from Aspen Database (Tsai *et al.* 2011). Suitable primers designed using Oligo Explorer were checked for similar melting temperature (*T*_m_), primer dimers and primer loops by Oligo Analyzer (Gene Link, Hawthorne, NY, USA; http://www.genelink.com/). Based on multiple sequence alignments done with program BioEdit (version 7.2.5; Hall 1999), the best gene-specific primer pairs were selected for *PtaRR3* and *PtaRR10*. Attempts to design gene-specific primers for other *PtaRR* type-A genes were not successful. We used three reference genes, among which Potri.005G074900 (similar to ubiquitin-specific protease4 [*UBI*]) was chosen according to Czechowski *et al.*, (2005) and newly tested for poplar (Wildhagen *et al.* 2017). The primer sequences for the *PtaRR* genes and reference genes [orthologs of Potri.019G006700 (*Actin*), Potri.005G074900 (similar to ubiquitin-specific protease4 [*UBI*]) and Potri.001G047200 (PPR repeat [*PPR*])] have been compiled in Table 1.

**Table 1.** Primer sequences used for quantitative real-time PCR.

Name	Potri. ID	Primer 5′-3′ direction	Primer efficiency	Reference
PtaRR3_fw PtaRR3_rev	Potri.002G082200	GCAACAGAAGGAAGGGGAAG GGACGACGAAGAAGAAGAGG	1.909	This study
PtaRR10_fw PtaRR10_rev	Potri.015G070000	CTTGGCTCGTATTGATAGGTGT TGCTCTCCTTCGCCTCCC	1.906	This study
Actin_fw Actin_rev	Potri.019G006700	TGGTGGTTCCACTATGTTCC TGGAAATCCACATCTGCTGG	1.876	Janz *et al.* (2012)
UBI_fw UBI_rev	Potri.005G074900	ACCAATGAGACAAGGTGCTT CTTTTGGGCTTCTTGCAAAC	1.899	Wildhagen *et al.* (2017)
PPR_fw PPR_rev	Potri.001G047200	GGCTGAGGAATGTCGAATGG AGAACGCAACATCATGGAAACC	1.902	Kavka and Polle (2016)

For the quantitative real-time PCR (qRT–PCR), the total reaction volume of 20 µL consisted of 10 µL SYBR Green I Master kit (Roche Diagnostics, Mannheim, Germany), 2 µL each of forward and reverse primer (10 µM), 1 µL nuclease-free water and 5 µL cDNA solution (1:10 dilution). The steps involved pre-incubation at 95 °C for 5 min, 55 cycles of amplification including a denaturation step at 95 °C for 10 min, an annealing step at 58 °C (for reference genes), 49 °C (*PtaRR3*) or 52 °C (*PtaRR10*) for 10 s and an elongation step at 72 °C for 15 s (for *PtaRR3*) or 20 s (for reference genes and *PtaRR10*). Each biological sample (*n* = 4) was measured in three technical replicates with the LightCycler 480® (Roche Diagnostics, Mannheim, Germany).

Melting curves (95 °C 5 s, 65 °C 1 min to 97 °C with an increase of 0.11 °C s^−1^) were generated to analyse primer specificity using the LightCycler 480® software. Raw data obtained were converted using LC480 conversion (version 2014.1; www.hartfaalcentrum.nl/index.php?main=files&sub=LC480Conversion) and imported to LinRegPCR (version 2016.0; Ruijter *et al.* 2009) to calculate the primer efficiency for each gene. The mean efficiency for each primer pair was calculated over all samples after baseline subtraction. For calculating the quantification cycle (*Cq*) values, a fluorescence threshold of 3.741 was used for all genes. Relative expression (*R*) of each sample in relation to three reference genes was calculated using the formula:

$$R=\frac{\sqrt[{3}]{\left[E^{Cq}{\text{(Rf}}_{1})\times E^{Cq}{\text{(Rf}}_{2})\times E^{Cq}{\text{(Rf}}_{3}\text{)}\right]}}{E^{Cq}\text{(GI)}}$$

where *E* is the primer efficiency for the gene, *Cq* is the quantification cycle value, *E*^*Cq*^(Rf_*i*_) is the primer efficiency and *Cq* for the reference gene *i*, and *E*^*Cq*^(GI) is the primer efficiency and *Cq* for the gene of interest (Hellemans *et al.* 2007).

### Statistical analyses

Statistical analyses were performed using the free statistical software R (version 3.1.1; R Core Team 2014). Three-way ANOVA was conducted for soil moisture, predawn leaf water potential, net photosynthesis rate and transpiration rate with plant lines (transgenic reporter lines and WT), drought and time as fixed factors and plant number as random factor to account for repeated measurements. We tested main effects (plant line, time, drought) and interactions. Two-way ANOVA was conducted for plant height, stem diameter, area per leaf, whole-plant leaf area, dry biomass of leaves, stem, coarse and fine roots, fine root to coarse root dry mass ratio and dry mass of leaves shed during treatment with plant lines (transgenic reporter lines and WT) and drought as factors. Main effects (drought, plant line) and interactions were tested.

For relative expression data obtained from qRT–PCR experiments, a three-way ANOVA was done for each gene with plant line, drought and tissue as main factors and their interactions. Because plant line had no effect, the factor line was excluded and for homogeneous subsets a *post hoc* Tukey HSD was conducted with treatment and tissue as factors. One-way ANOVA was conducted for the relative expression values of *PtaRR3* and *PtaRR10* with gene as factor.

Normality and homogeneity of variance were checked visually using residual plots and data were transformed logarithmically (log_2_) if required. Data shown are mean ± SE. Means were considered to be significantly different with a *P*-value ≤ 0.05.

## Results

### Physiological responses and growth performance of well-watered and drought-stressed WT and poplar reporter lines

**Figure 1.**
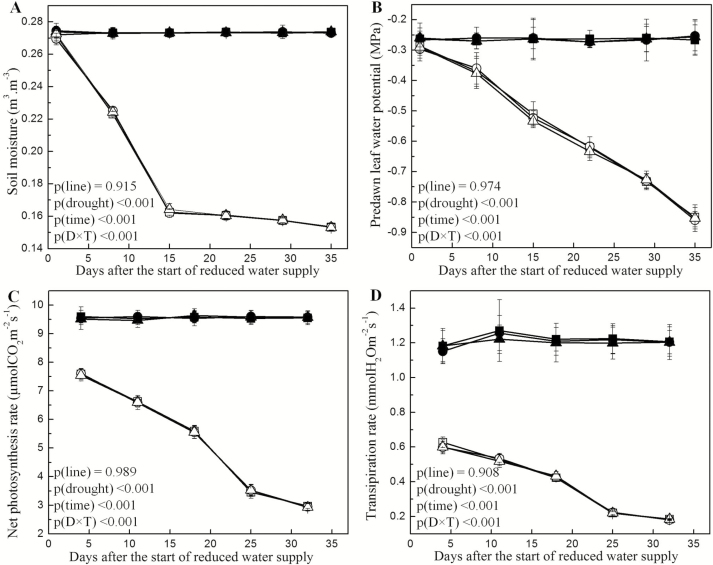
Effect of drought on the physiological parameters of *Populus × canescens* reporter lines and WT during the treatment period. The graphs represent soil moisture (A), predawn leaf water potential (B), net photosynthesis rate (C) and transpiration rate (D) during the treatment period. Data are means ± SE (*n* = 7 for soil moisture content, *n* = 3 for predawn leaf water potential and *n* = 5 for net photosynthesis rate and transpiration rate). The *P*-values as detected by repeated-measures three-way ANOVA are provided. Statistical details are shown in **Supporting Information—Table S1**. The WT, lines 64 and 80 are represented using square, circle and upright triangle, respectively. Closed symbols represent well-watered and open symbols represent drought-treated plants. When error bars are not visible, they were smaller than the symbols.

The reduced water supply resulted in significantly reduced soil moisture in comparison with that in the pots of well-watered plants (Fig. 1A). Predawn leaf water potential (Fig. 1B), net photosynthesis (Fig. 1C) and transpiration rates (Fig. 1D) were also significantly reduced under drought but did not show any differences between WT poplars and the GUS reporter lines. All parameters showed significant changes with the factor ‘time’ indicating increasing drought stress. Significant interactions were found between the main factors ‘drought’ and ‘time’ because the controls remained unchanged, whereas soil moisture, predawn leaf water potentials, photosynthesis and transpiration declined significantly in response to the reduced water supply (Fig. 1).

**Figure 2.**
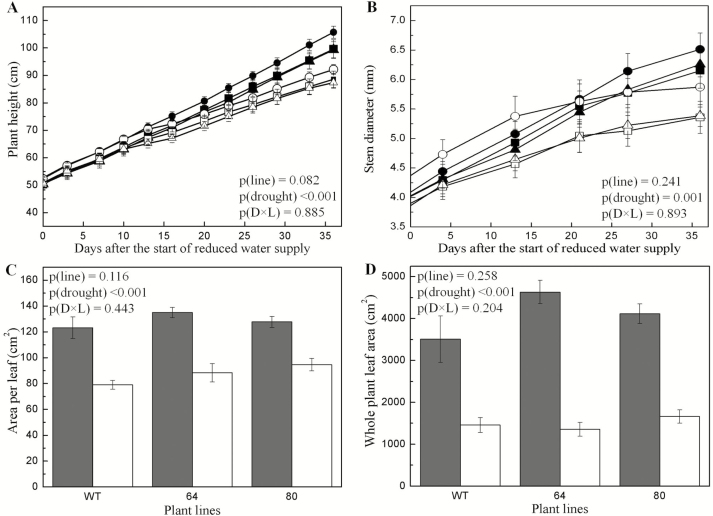
Effect of drought on the morphological parameters of *Populus × canescens* reporter lines and WT during the treatment period. The graphs represent plant height (A), stem diameter (B), area per leaf (C) and whole plant leaf area (D). Data are means ± SE (*n* = 7). The *P*-values as detected by a two-way ANOVA are provided. Statistical details are shown in **Supporting Information—Table S1**. In graphs (A) and (B), WT, lines 64 and 80 are represented using square, circle and upright triangle, respectively. Closed symbols represent well-watered and open symbols represent drought-treated plants. In the bar graphs (C) and (D), well-watered plants are represented as grey bars while drought-treated plants are represented as open bars.

**Figure 3.**
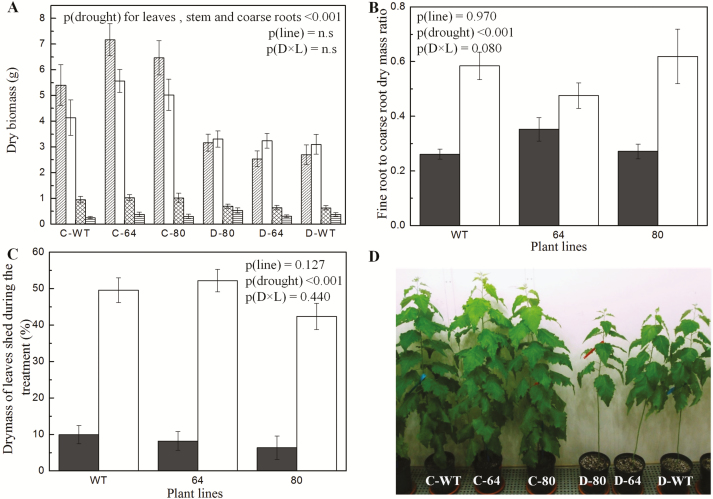
Biomass (A), fine root to coarse root ratios (B), fraction of shed leaves (C) and photo of representative *Populus × canescens* reporter lines and WT plants before harvest (D) of well-watered and drought-treated plants. In graph (A) bars correspond to leaves (hatched), stem (white), coarse roots (crossed) and fine roots (hatched horizontally). Data are means ± SE (*n* = 7 per line and treatment). The *P*-values as detected by a two-way ANOVA are provided. Statistical details are shown in Supporting Information—Table S1. In graph (A) for fine roots, *p*(drought) was not significant. ‘C’ stands for control (well-watered) and ‘D’ stands for drought-treated.

The drought-stressed plants were characterized by strongly reduced plant heights (Fig. 2A), stem diameters (Fig. 2B), area per leaf (Fig. 2C) and whole-plant leaf area (Fig. 2D) when compared to well-watered plants. Dry biomass of the leaves [*p*(drought) < 0.001], stem [*p*(drought) < 0.001] and coarse roots [*p*(drought) = 0.001] of the drought-treated plants was significantly lower than that of the well-watered plants while no drought effects were found for fine root biomass [*p*(drought) = 0.105] (Fig. 3A). Fine root to coarse root dry mass ratio showed a significant increase under drought (Fig. 3B). Drought led to significantly increased leaf shedding (Fig. 3C). Neither significant line effects nor drought × line interaction effects were observed for any of the plant growth parameters (Figs 2 and 3A–D).

### Cytokinin activity in tissues of well-watered and drought-stressed poplars traced by *ARR5::GUS* reporter lines

**Figure 4.**
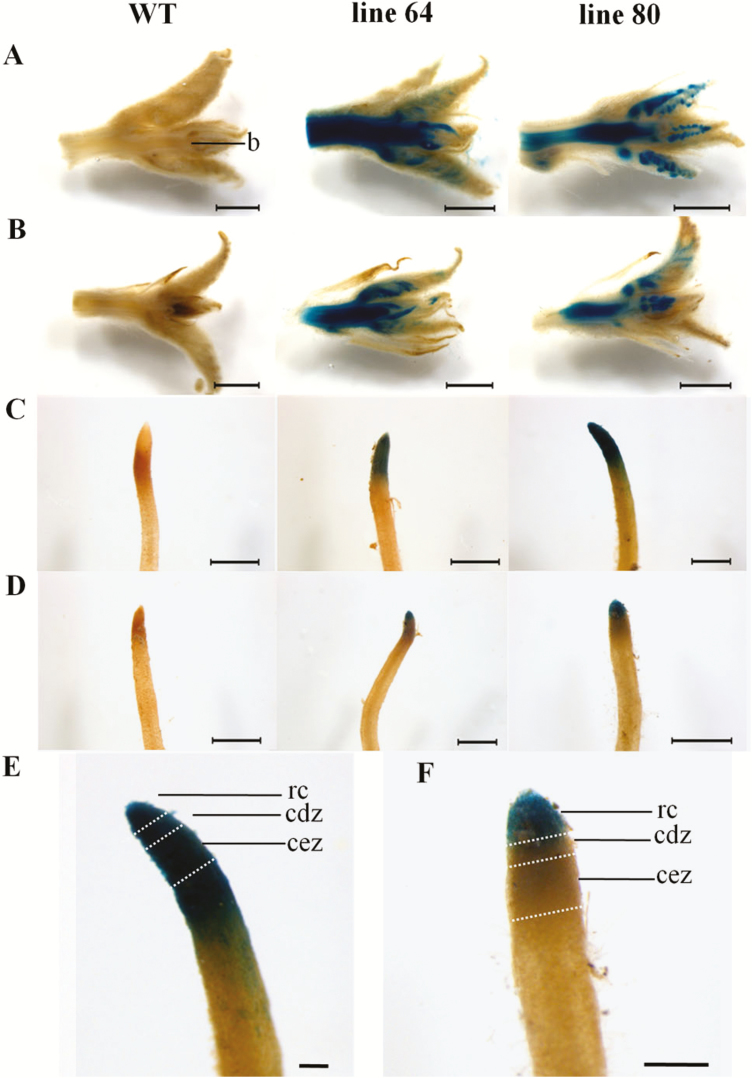
*ARR5::GUS* activity observed in shoot and root apical meristems of *Populus × canescens* reporter lines and WT under well-watered and drought conditions. Representative images of *n* = 7 replicates per line and treatment are shown. Rows (A) and (B) show shoot apices (b with indicating the apex base) from well-watered and drought-treated plants, respectively. Rows (C) and (D) show fine roots from well-watered and drought-treated plants, respectively. The images (E) and (F) show a closer look at the tip region of fine root from line 80 under well-watered and drought conditions, respectively. The root zones rc, root cap; cdz, cell division zone; and cez, cell elongation zone labelled after focussing through the tissue. Scale bar = 2 mm for rows (A) and (B); 1 mm for rows (C) and (D); and 250 µm for images (E) and (F).

*ARR5::GUS* activity was examined in shoot apices, young leaves, leaf scars, stem cross-sections from three positions, i.e. top, middle and bottom, and root tips. Poplar reporter lines 64 and 80 showed *ARR5::GUS* activity in all tissues investigated. In the shoot apices collected from well-watered plants, *ARR5::GUS* activity was detected in the leaf primordia and shoot apex base (Fig. 4A). The *ARR5::GUS* activity extended further into the pith region. Shoot apices collected from the drought-treated plants (Fig. 4B) showed localization and intensity of *ARR5::GUS* activity similar to that of controls (Fig. 4A). In contrast to the shoot apex meristem, the apical meristem of fine root tips displayed notable difference between well-watered and drought-stressed plants (Fig. 4C and D). In the fine roots from well-watered plants, strong *ARR5::GUS* activity was present in the root cap region, in the cell division zone and also in the elongation zone (Fig. 4E). Thereafter, in the more mature root section the staining showed a gradual reduction (Fig. 4E). In the fine roots of drought-treated plants, *ARR5::GUS* activity was observed only in the root cap region (Fig. 4F). After this region, a sharp drop in the staining intensity was found (Fig. 4F), unlike the gradual fading of the colour in roots of well-watered plants (Fig. 4E).

**Figure 5.**
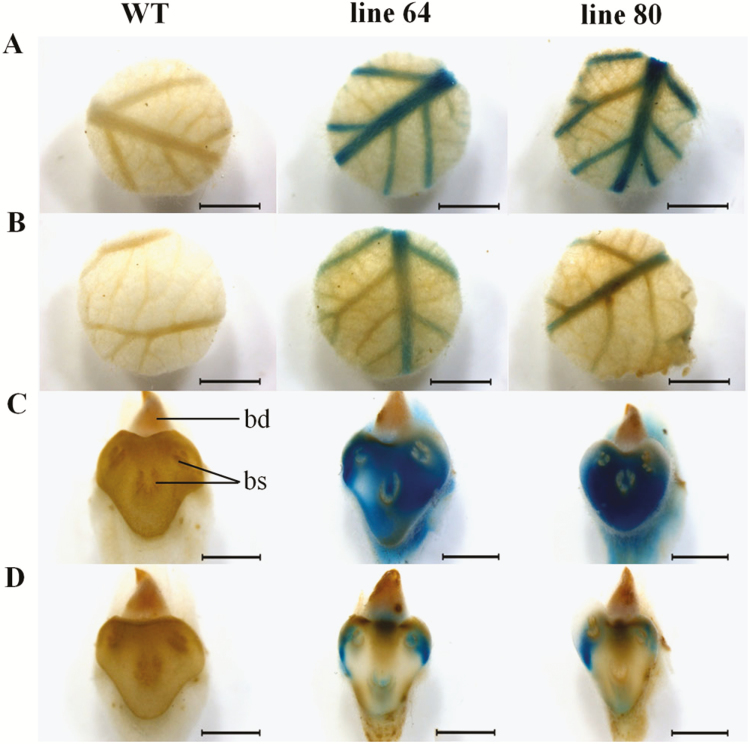
*ARR5::GUS* activity observed in leaf disks and leaf scars of *Populus × canescens* reporter lines and WT under well-watered and drought conditions. Representative images of *n* = 7 replicates per line and treatment are shown. Rows (A) and (B) show leaf disks from well-watered and drought-treated plants, respectively. Rows (C) and (D) show leaf scars from well-watered and drought-treated plants, respectively. Here, abbreviations indicate: bd, lateral bud; and bs, bundle scar. Scale bar = 2 mm.

The young leaves from the well-watered plants showed a strong *ARR5::GUS* activity mainly in the primary and secondary veins (Fig. 5A). Young leaves from drought-stressed plants also showed *ARR5::GUS* activity in the primary and secondary veins (Fig. 5B) but the staining intensity was weaker compared to that observed in the well-watered plants. Mature leaves did not show any GUS staining (not shown). Leaf scars from the well-watered plants showed intense *ARR5::GUS* activity (Fig. 5C), whereas those from the drought-treated plants showed weak *ARR5::GUS* activity (Fig. 5D). Here, the *ARR5::GUS* activity was detected in the bundle scars (markings within a leaf scar where bundles of vascular tissue had once connected the leaf and stem) or in close proximity to them.

**Figure 6.**
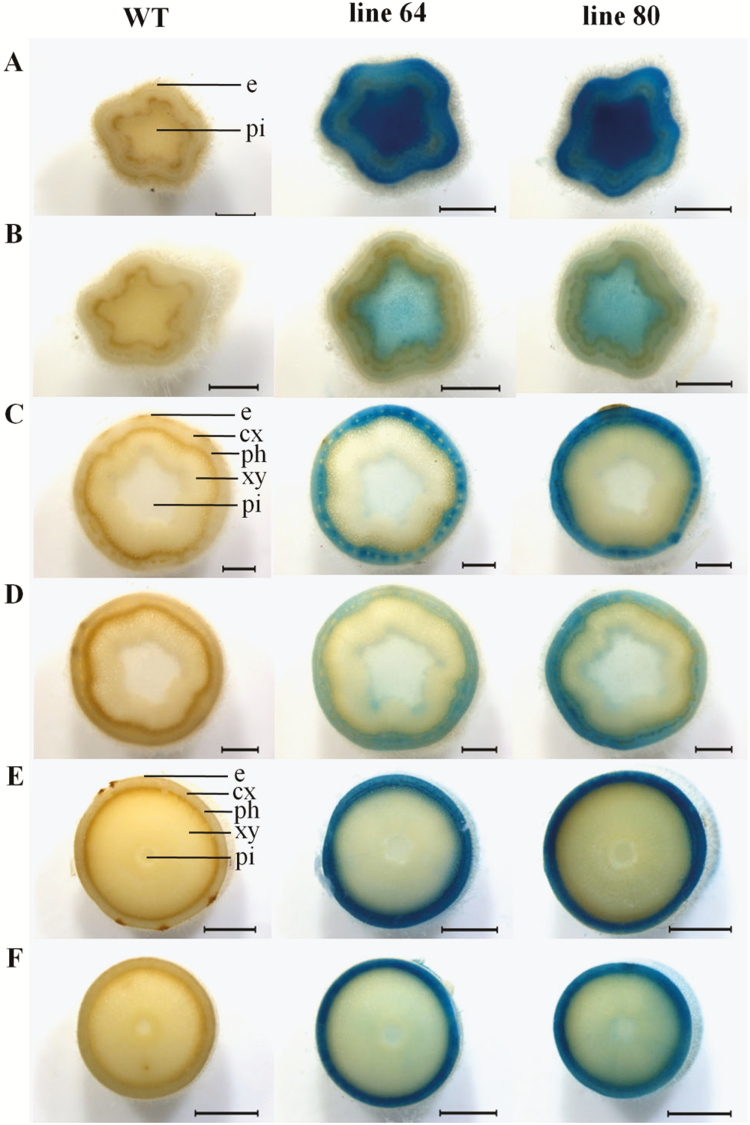
*ARR5::GUS* activity observed in different stem sections of *Populus × canescens* reporter lines and WT under well-watered and drought conditions. Representative images of *n* = 7 replicates per line and treatment are shown. Rows (A) and (B) show stem top from well-watered and drought-treated plants, respectively. Rows (C) and (D) show stem middle from well-watered and drought-treated plants, respectively. Rows (E) and (F) show stem bottom from well-watered and drought-treated plants, respectively. Here, e, epidermis; cx, cortex; ph, phloem; xy, xylem; and pi, pith. Scale bar = 1 mm for rows (A–D), and 2 mm for rows (E) and (F).

In stem cross-sections of the stem elongation zone, close to the apex, strong *ARR5::GUS* activity was discovered in the pith tissue of well-watered plants (Fig. 6A), whereas drought-stressed plants showed a weaker *ARR5::GUS* activity in the pith (Fig. 6B). Further down the stem, strong *ARR5::GUS* activity, as present in the pith in the stem top region, was not detected (Fig. 6C–F). Cross-sections of all positions studied along the stem of well-watered plants showed strong *ARR5::GUS* activity in the bark (Fig. 6A, C and E). In contrast, the drought-treated plants displayed a very weak staining in the cortex region of stem top (Fig. 6B), and increasing staining intensities in the bark towards the middle and bottom position of the stem (Fig. 6D and F).

### Cytokinin activity in different cell types of stem tissues in well-watered and drought-stressed poplars traced by *ARR5::GUS* reporter lines

**Figure 7.**
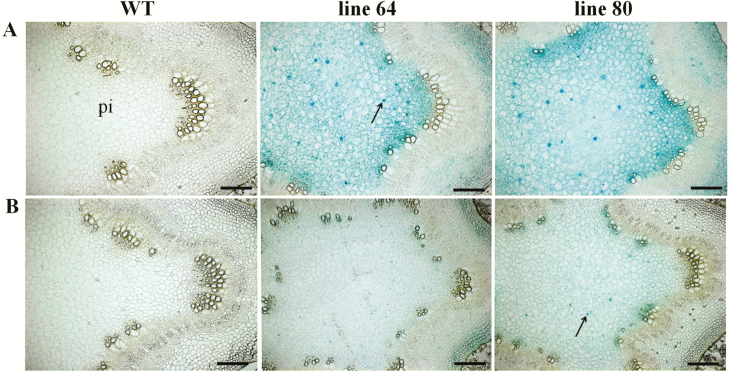
*ARR5::GUS* activity observed in parenchymatous cells of pith and cortex region of the stem top section of *Populus × canescens* reporter lines and WT under well-watered and drought conditions. Representative images of *n* = 7 replicates per line and treatment are shown. Rows (A) and (B) show stem top sections from well-watered and drought-treated plants, respectively. Here, ‘pi’ represents pith region of the stem top section. The arrows show the very active cells found in the pith. Scale bar = 200 µm.

**Figure 8.**
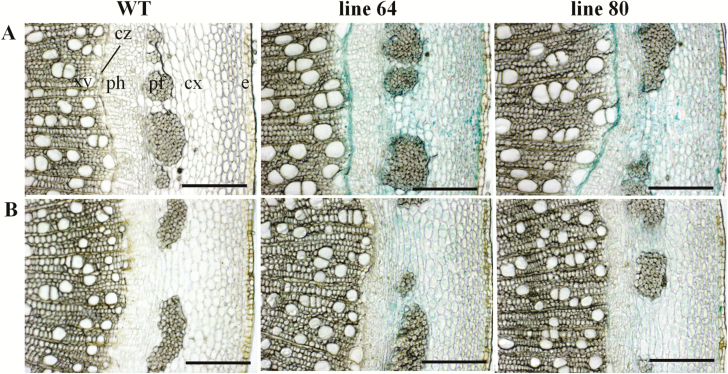
*ARR5::GUS* activity observed in the cambial and cortical cells of stem middle section of *Populus × canescens* reporter lines and WT under well-watered and drought conditions. Representative images of *n* = 7 replicates per line and treatment are shown. Rows (A) and (B) show stem middle sections from well-watered and drought-treated plants, respectively. Here, e, epidermis; cx, cortex; pf, phloem fibres; ph, phloem; cz, cambial zone; and xy, xylem. Scale bar = 200 µm.

**Figure 9.**
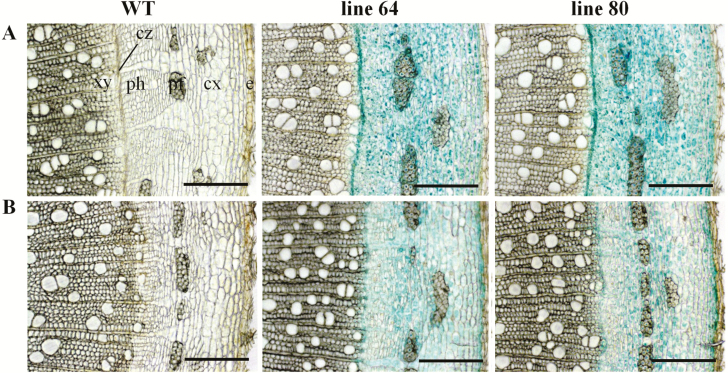
*ARR5::GUS* activity observed in the cambial, phloem and cortical cells of stem bottom section of *Populus × canescens* reporter lines and WT under well-watered and drought conditions. Representative images of *n* = 7 replicates per line and treatment are shown. Rows (A) and (B) show stem bottom sections from well-watered and drought-treated plants, respectively. Here, e, epidermis; cx, cortex; pf, phloem fibres; ph, phloem; cz, cambial zone; and xy, xylem. Scale bar = 200 µm.

Stem cross-sections were further analysed at higher magnification to examine the *ARR5::GUS* activity in different cell types. In the stem elongation zone of the well-watered plants, strong *ARR5::GUS* activity was confirmed in the pith region (Fig. 7A). *ARR5::GUS* activity was also seen in the cortex and in the outer pith region (perimedullary zone) associated with the primary xylem. A few active cells which showed strong *ARR5::GUS* activity compared to the other pith cells were also noted here (Fig. 7A). In the stem top section of the drought-treated plants, weak *ARR5::GUS* activity was confirmed for the pith and in the cortex regions (Fig. 7B). A few distinct, particularly active cells were also present in the pith of drought-stressed plants, but with weaker staining intensity and less in number compared to the well-watered plants (Fig. 7B). In the stem middle section from the well-watered plants, a strong *ARR5::GUS* activity was noted in the cambial zone that was adjacent to first row of developing xylem (Fig. 8A). The staining was easily notable as a continuous line (Fig. 8A). In the bark, cell layers just below the epidermis and cells between the phloem fibre groups showed higher *ARR5::GUS* staining than other cells in the cortex region. In the phloem region (below the cortex), *ARR5::GUS* activity was not so prominent. In the corresponding section of the drought-treated plants, the strong staining of the cambial cell layer disappeared (Fig. 8B). Otherwise, the staining pattern was similar to that of well-watered plants but with less intensity (Fig. 8B). In the sections from the bottom of the stem of the well-watered plants, strong *ARR5::GUS* activity was also noted in the cambial zone (Fig. 9A) as in the stem middle. The cortex and phloem region showed an even and intense staining. No *ARR5::GUS* activity was detected in the xylem region. In the stem bottom section of the drought-stressed plants, the staining pattern in cortex and phloem was similar to that found in well-watered plants but with slightly lower intensity (Fig. 9B). At the stem bottom of drought-stressed plants, *ARR5::GUS* activity was observed in the cambial zone, but as a broken and fading line unlike the clear continuous line that was observed in the stem of well-watered plants.

### Expression analysis of *PtaRR3* and *PtaRR10* in well-watered and drought-stressed poplars

**Figure 10.**
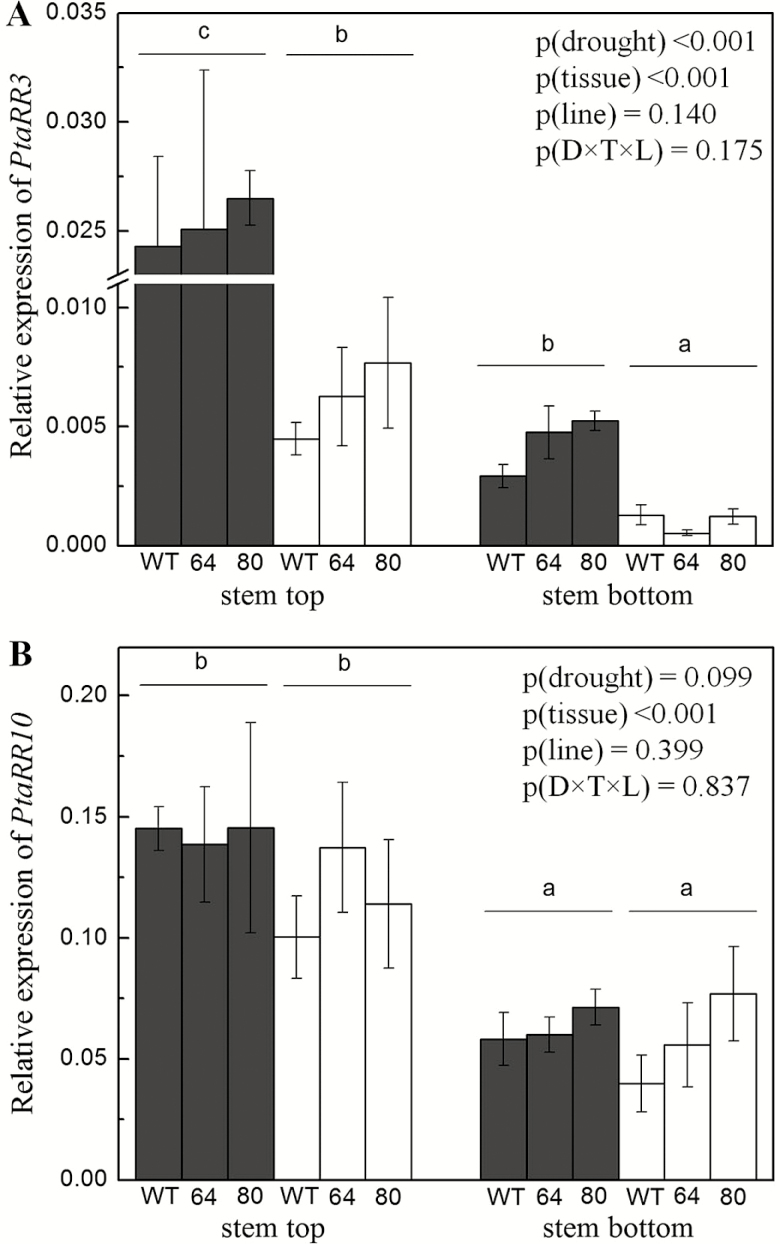
Effect of drought on the relative expression levels of PtaRR type-A genes, *PtaRR3* and *PtaRR10* in debarked tissues from the stem top and stem bottom. The graph represents effect of drought on the tissue-specific expression of *PtaRR3* and *PtaRR10* in relation to three reference genes, *Actin*, *UBI* and *PPR*. Data are means ± SE (*n* = 4 biological replicates per treatment, tissue and line). The *P*-values are given as detected by a three-way ANOVA. Statistical details are shown in Supporting Information—Table S1. Well-watered plants are represented as grey bars and drought-treated by open bars. The positions of stem top and bottom correspond to those shown in Fig. 6A and B and in Fig. 6E and F, respectively.

*PtaRR3* was higher expressed in the debarked samples from the stem top than in those from the stem bottom (Fig. 10A). *PtaRR3* was further significantly higher expressed in tissues of well-watered than those of drought-stressed poplars (Fig. 10A) reflecting the GUS staining pattern of the stem tissues under drought.

*PtaRR10* was also significantly higher expressed in debarked samples from the stem top than in those from the stem bottom, similar to the expression of *PtaRR3* (Fig. 10B). But the overall expression level of *PtaRR10* was significantly higher than that of *PtaRR3* (*P* < 0.001; Fig. 10A and B). The expression pattern of *PtaRR10* in well-watered poplars corresponded to the GUS activity pattern observed in these tissues (Fig. 6A, B, E and F). In contrast to the strong decrease observed for the GUS staining in response to drought treatment in the top region of the stem (Fig. 6B), only marginal reductions in *PtaRR10* gene expression were found in the top as well as in the bottom of the stem (Fig. 10B).

Neither the *PtaRR3* nor the *PtaRR10* expression levels exhibited differences among the WT or reporter lines. Furthermore, no interactions among the main factors drought treatment, tissue or line were observed (Fig. 10A and B).

## Discussion

### Performance of the WT and ARR5 lines under well-watered and drought-stressed conditions

Growth and morphology of the *ARR5::GUS* poplar reporter lines 64 and 80 showed no differences compared to that of WT poplars. This observation is in agreement with previous studies on the performance of the *ARR5::GUS* reporter lines (Paul *et al.* 2016) and underpins that the transformation did not affect any crucial growth-related process. Under drought stress, WT and reporter plants showed growth decline and leaf shedding, which is typical for poplars subjected to water limitations (Polle *et al.* 2006; Fischer and Polle 2010). It should be noted that poplars in this experiment were very sensitive to desiccation because they shed a large fraction of their leaves although the stress was very moderate with predawn leaf water potentials above −0.9 MPa throughout the whole time course of reduced water supply. This response may be a survival strategy because it reduces the area for transpiration and, thus, overall water consumption (Fischer and Polle 2010). In agreement with this suggestion, Hennig *et al.* (2015) showed that poplar genotypes with early leaf shedding under drought exhibited higher survival rates than those with longer leaf retention.

### Cytokinin activity in apical meristems, roots and leaves under drought stress

Cytokinins are positive regulators of shoot meristem and negative regulators of root meristem activity (Werner *et al.* 2003). Here, the pattern and intensity of staining of GUS activity in the shoot apices of well-watered was similar to that of drought-treated plants and not reduced as in the root tips (Fig. 4A and B). Root tips are the major biosynthetic sites of cytokinins (Dieleman *et al.* 1997; Miyawaki *et al.* 2004; Aloni *et al.* 2005) from where these phytohormones are transported with the xylem sap to the above-ground tissues (Aloni *et al.* 2005). The reduced staining in the root tips from the drought-treated plants when compared to the well-watered plants observed in this study could be due to either a decrease in cytokinin biosynthesis or increased degradation but apparently had no physiological consequence on the shoot apical cytokinin activity. Our results suggest that the cytokinin activity was kept up in the buds under drought stress, most likely because cytokinins have an important role in protecting and maintaining the shoot apical meristem (Werner *et al.* 2001).

The growth inhibitory effect of cytokinins in roots is due to their negative effects on cell division of the meristematic cells and also on the expansion of cells in the cell elongation zone of the root (Kieber and Schaller 2014). For instance, in cytokinin-deficient transgenic *Arabidopsis* plants, primary root growth increased because of an enhancement in the number of dividing cells in the root meristematic region without altering the cell division rate (Werner *et al.* 2003). The decline in cytokinin activity in the root tips of drought-stressed poplars in comparison with those of the well-watered plants (Fig. 4E) is, thus, expected to stimulate root growth. In agreement with this assumption, a significant increase in the fine root to coarse root dry mass ratio was noted here (Fig. 3B). Obviously, the resource allocation under drought was in favour of the fine root production. An enhanced fine root production is required for water uptake from deeper soils and forms a part of the drought avoidance mechanisms of poplars (Brunner *et al.* 2015).

In young poplar leaves, *ARR5::GUS* activity was present in the main veins, in agreement with the vascular transport of cytokinins (Aloni *et al.* 2005; Bishopp *et al.* 2011). In leaves, cytokinins play an important role in determining leaf size by driving the cell division cycle at a normal speed and attaining the required number of cell divisions (Werner *et al.* 2001). Leaves of drought-stressed plants contain decreased cytokinin contents (Pospíšilová *et al.* 2000). Here, the stronger GUS activity in the leaves from well-watered plants (Fig. 5A) corresponded to the stronger promotion of leaf area formation in the controls compared to the drought-treated plants (Fig. 2C and D). This finding is important because leaf area is a strong predictor for stem biomass production (Ridge *et al.* 1986; Barigah *et al.* 1994; Bartelink 1997).

Cytokinins inhibit leaf senescence (Gan and Amasino 1996) and therefore may be involved in regulating leaf shedding. So far, only the involvement of auxin and ethylene in this process has been studied in a tissue-specific manner (Jin *et al.* 2015). Our data propose a role of cytokinins in this developmental step because the cytokinin activity was high all over the leaf scars of the well-watered plants and decreased in the drought-treated plants (Fig. 5C and D). Leaf shedding resulted in reduction of whole-plant leaf area and water consumption. The reduced water demand may thereby eventually enable poplar survival under extended drought (Munné-Bosch and Alegre 2004; Hennig *et al.* 2015). Drought-induced leaf shedding starts at the bottom with the older leaves and moves up the stem with prolonged drought. An interesting question for future research is whether the transport from roots in the xylem and preferential supply of cytokinins to the stem apex contributes to this pattern.

### Cytokinin activity in relation to stem growth under drought

During primary vascular development cytokinins regulate both cell proliferation and differentiation but during the phase of secondary growth they mainly regulate cell proliferation (Nieminen *et al.* 2008; Sorce *et al.* 2013). Here, we localized cytokinin activity along the stem axis and found changes in tissue localization when comparing zones of primary and secondary growth. In well-watered poplars cytokinin activity was high in the pith tissue in top of the stem and declined in xylem towards the bottom where secondary growth took place. This pattern was similar to that reported previously for outdoor-grown poplars in summer with high staining in the pith tissue in the stem elongation zone (Paul *et al.* 2016). The staining pattern was further congruent with a relatively high cytokinin content in the active phloem cells along with cambial cells, a cytokinin maximum in the developing phloem cells and a very low cytokinin concentration in the developing as well as lignified xylem tissues across *P. trichocarpa* cambial zone found by Immanen *et al.* (2016). The observed *ARR5::GUS* activity also corresponded to the expression levels of poplar type-A RR genes, *PtaRR3* and *PtaRR10*, which were high in the stem top and low in secondary xylem. High *ARR5::GUS* activities in the pith, especially next to the young differentiating vascular tissues (Fig. 7A and B), were notable and we can only speculate about their function. Cytokinins are important for determining vascular cell-type identities except protoxylem (Mähönen *et al.* 2000; Hutchison *et al.* 2006; Yokoyama *et al.* 2007; Argyros *et al.* 2008). Furthermore, the pith also contains high auxin activities monitored by *GH3::GUS* (Teichmann *et al.* 2008) and *DR5::GUS* auxin reporter poplars (Chen *et al.* 2013). Therefore, it is possible that the pith forms a reservoir for auxin and cytokinins, whose supply may control growth processes. Since drought resulted in a decline in *ARR5::GUS* activity, but not in changes in cell identities, we speculate that changes in cytokinin levels in the pith may be involved in regulating the growth speed, but not differentiation. The observation of very active cells in the pith in both well-watered and drought-treated plants is novel and suggests functional differentiation of parenchyma cells but the relevance of these cells is not clear.

Cytokinins control wood quantity and quality by enhancing the sensitivity of the cambial cells to auxin (Aloni 1991; Aloni 2001). Immanen *et al.* (2016) reported a relatively high cytokinin level in the dividing cambial cells of *P. trichocarpa*, whereas a reduced cambial cytokinin concentration led to compromised radial growth in transgenic *Populus* (Nieminen *et al.* 2008). The intense *ARR5::GUS* activity in the cambium in the stem portions with strong secondary growth (Figs 8 and 9) and the low, irregular staining under drought when radial growth was low strongly supported earlier findings that cytokinins act as main regulators of cambial activity in poplars are important regulators of cambial activity in growing poplars (Matsumoto-Kitano *et al.* 2008; Nieminen *et al.* 2008). Nieminen *et al.* (2008) noted an expression peak for cytokinin receptor genes along with a high expression of *PtRR7*, a type-A RR gene family member, in the dividing cambial cells of poplar. Here, we determined *PtaRR3* and *PtaRR10* expression levels for wood extracts, where according to the visual assessment of staining, the expression levels were lower than in stem top tissues. Since wood tissues still contain living and immature cells after debarking (Teichmann *et al.* 2008), the presence of low type-A RR gene expression and a decline in response to drought was expected. The reason for the observed unexpected behaviour of *PtaRR10* expression, the ortholog of ARR5 in poplar, is probably due to differences in the promoter structures. Divergent genome-wide drought responses have also been reported between two genotypes of *Populus canadensis* (Cohen *et al.* 2010) indicating specificity of drought acclimation. Here, drought-induced decline occurred only for *PtaRR3* and not for *PtaRR10*, suggesting divergent roles for these genes in drought-induced cytokinin signalling. Since poplars contain 11 type-A RR genes, more work needs to be done to better understand the function of individual members of this gene family in mediating tissue- and cell-specific cytokinin responses in wood formation.

## Conclusions

Drought resulted generally in reduced cytokinin activity, except in root and stem apices. The tissue- and cell-specific localization is attributable to different functions of cytokinins: in the primary meristems high cytokinin activities under drought support a role of this phytohormone in meristem maintenance (Leibfried *et al.* 2005). In the cambium, a secondary meristem, drought resulted in a drastic loss of cytokinin activity suggesting that here the regulation of cell proliferation is predominant. High cytokinin activity was also detected in the undifferentiated ground tissue of the apical stem pith of well-growing control poplars, whereas drought-induced growth reduction was accompanied by decreased cytokinin activities at this localization, suggesting a control function of cytokinins in the apical pith for stem elongation. Since cytokinins suppress root growth (Kieber and Schaller 2014), the reduced cytokinin activity in roots under drought, except the tip meristem, is compatible with the relative enhancement of fine root biomass and, thus, may contribute decisively to avoid early water deprivation. The localization and changes in staining patterns of cytokinin activity in leaf veins and leaf scars further support that down-regulation of cytokinin activities may contribute to poplar drought avoidance since leaf area was diminished by shedding of existing leaves and formation of smaller leaves under drought. These morphological changes are known to enhance plant survival under drought.

## Sources of Funding

This work was supported by the European Union’s Seventh Programme for research, technological development and demonstration in the framework of WATBIO (Development of improved perennial non-food biomass and bioproduct crops for water-stressed environments), which is a collaborative research project (grant number 311929). The European Commission granted a PhD scholarship to S.P. in the Erasmus Mundus India4EU II programme. This publication reflects the views only of the authors, and the European Commission cannot be held responsible for any use which may be made of the information contained therein.

## Contributions by the Authors

S.P. conducted the climate chamber experiment, analysed all data and wrote the manuscript. H.W. supervised experiments, analysed data and commented on the manuscript. D.J. analysed bioinformatics data and commented on the manuscript. A.P. designed the experiments, supervised the research, discussed and analysed data and wrote the manuscript.

## Conflict of Interest

None declared.

## Supporting Information

The following additional information is available in the online version of this article—

**Table S1.** Details of statistical analyses showing degrees of freedom (df), *F* values and *P*-values for each ANOVA conducted. Significant differences at *P* < 0.01** and *P* < 0.001*** are indicated.

## Supplementary Material

Supporting Information
